# The impact of the COVID-19 pandemic on public trust in science

**DOI:** 10.1371/journal.pone.0328075

**Published:** 2025-09-29

**Authors:** Alain Abi-Rizk

**Affiliations:** Department of Agricultral and Food Engineering, School of Engineering, Holy Spirit University of Kaslik, Jounieh, Lebanon; Lahore Medical and Dental College, PAKISTAN

## Abstract

Public trust in science plays a critical role in shaping attitudes toward health policies, technological advancements, and societal progress. The COVID-19 pandemic profoundly influenced science communication, governmental policies, and public perceptions of scientific credibility. This study examines shifts in trust in science before and after the pandemic using survey data from 10,000 respondents across multiple demographic groups. Results indicate that respondnatds from North America and Europe Experienced an increase in trust where as Africa and South America Witnessed a decline in trust. Males exhibited a greater decline in trust than females (p = 0.038), and undergraduate degree holders showed the largest decrease (p = 0.001). Notably, individuals who relied on independent researchers for scientific information exhibited a slight increase in trust, whereas those who consumed traditional media experienced the largest decline (p < 0.001). These findings highlight the complex dynamics of science trust and the importance of targeted science communication strategies to mitigate erosion in confidence.

## Introduction

Trust plays a crucial role in public health emergencies, where adherence to expert recommendations can determine the success of response strategies. To understand the dynamics of public trust, this study draws on the Cultural Cognition Theory, which suggests that individuals interpret scientific information in a way that aligns with their group values and cultural identities. [1,2]. It determines responses to medical guidance, climate policies, and new technologies [3]. A high level of trust is linked to adherence to expert recommendations [4], while declining trust contributes to vaccine hesitancy [5] and climate skepticism [6]. The COVID-19 pandemic was a crucial test for science communication, revealing both innovation (e.g., vaccine development) and challenges (e.g., misinformation, political polarization) [7]. Frequent revisions to public health guidelines, perceived inconsistencies, and political interference contributed to public skepticism [8].

While initial responses to the pandemic saw a surge in trust toward experts [9], conflicting information and media polarization later contributed to its decline [10]. Mistrust in vaccines stemmed not only from misinformation but also from preexisting skepticism toward scientific institutions [11].

This study quantifies trust shifts in science before and after the pandemic, examines variations based on demographic factors, and evaluates how media sources influenced public perceptions. The findings provide empirical insights into the long-term effects of COVID-19 on science trust and the role of information dissemination in shaping attitudes.

## Methods

A nationally representative survey of 10,000 adult respondents was conducted across over 25 countries on all inhabited continents. Quotas were set proportionally based on population size and internet access to ensure geographic diversity, between 02/10/2023 and 20/10/2024, stratified by age, gender, education level, and information sources. Prior to participation, each respondent provided informed consent by agreeing to a statement confirming that the survey would remain anonymous and be used for research purposes only. The study protocol was approved by the Research Ethics Committee (REC) of the Higher Center for Research (HCR) at USEK under the reference number HCR/EC 2023−057. Trust in science was measured on a Likert scale from 1 (low trust) to 10 (high trust). Data was collected via online panels. While this is a single-item measure, it was chosen for its simplicity in large-scale surveys. This approach balances depth and respondent engagement, though it limits multi-dimensional insight.

All statistical analyses were conducted using R (version 4.3) and Python (version 3.12). A significance threshold of α = 0.05 was applied to all inferential tests.

### Descriptive statistics

Initial data exploration involved computing the mean, median, and standard deviation of trust in science scores before and after the COVID-19 pandemic.

### Paired T-Test for change in trust

To assess whether the mean change in trust scores was statistically significant, a paired t-test was performed, accounting for individual-level differences before and after the pandemic. The test statistic was calculated as:


$$\begin{array}{c} \text{t}\;=\;\bar{\text{d}}\;/\;(\text{s}\text{d}\;/\;\sqrt{}\text{n}),\; \end{array}$$

where $\begin{array}{c} \bar{\text{d}} \end{array}$ represents the mean difference in trust scores, sd denotes the standard deviation of differences and n is the sample size.

Cohen’s d was computed to quantify the magnitude of the observed effect:


$$\begin{array}{c} \text{d}\;=\;\bar{\text{d}}\;/\;\text{s}\text{d},\; \end{array}$$

where values of 0.2, 0.5, and 0.8 indicate small, medium, and large effects, respectively.

### Demographic subgroup analysis

To explore variations in trust change across population subgroups, independent t-tests were conducted for binary categorical variables, One-way ANOVA was used to compare trust change across education levels and age groups, Post hoc Tukey’s HSD tests were applied to identify specific group differences where significant ANOVA results were observed, and Welch’s ANOVA was used when the assumption of variance homogeneity was violated.

### Correlation analysis

The relationship between age and trust change was examined using Pearson’s correlation coefficient:


$$\text{r}\;=\;\Sigma \;(\text{X}\;-\;\overset{―}{\text{X}})\;(\text{Y}\;-\;\overset{―}{\text{Y}})\;/\;\sqrt{}\;(\Sigma \;(\text{X}\;-\;\overset{―}{\text{X}}{)}^{2}\;\Sigma \;(\text{Y}\;-\;\overset{―}{\text{Y}}{)}^{2}),$$

where X represents age and Y represents trust change scores. A correlation coefficient close to zero indicates no meaningful relationship.

### Regression modeling

A multiple linear regression model was constructed to identify predictors of trust change:


$$\begin{array}{c} \Delta \text{\{Trust}\}=\beta_{0}+\beta_{1}(\text{\{Age}\})+\beta_{2}(\text{\{Gender}\})+\beta_{3}(\text{\{Education}\})+\beta_{4}\;(\text{\{Information}\}\;\text{\{Source}\})+\varepsilon ,\; \end{array}$$

where: βi are regression coefficients, and ε represents the error term.

The adjusted R² value was reported to evaluate the model’s explanatory power.

### Data visualization

All visualizations were generated using ggplot2 (R) and Matplotlib/Seaborn (Python), including Boxplots comparing trust scores before and after the pandemic, histograms illustrating the distribution of trust change, and scatter plots depicting correlations between continuous variables.

## Results

### Overall change in trust in science

The analysis revealed a small but statistically significant decline in public trust in science following the COVID-19 pandemic. The mean trust score before the pandemic was 5.58 ± 1.92, while the mean trust score after the pandemic was 5.55 ± 1.94, resulting in a net decrease of 0.03 points (95% CI: −0.05 to −0.01), which is statistically significant but negligible in practical terms (Cohen’s d = 0.02).

**Fig 1:**
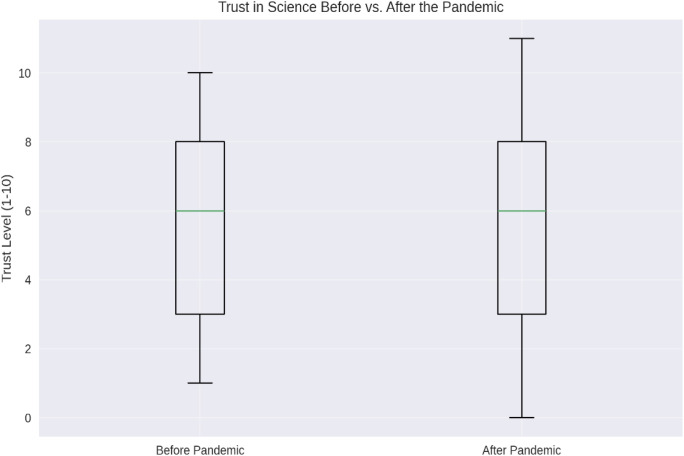
Trust levels before and after the COVID-19 pandemic (n = 10,000). Boxplot comparing self-reported trust in science before and after the pandemic using a 10-point Likert scale (1 = low trust, 10 = high trust). While the median remained stable, the overall distribution shows slight changes, reflecting subtle shifts in public perception. A paired t-test revealed a statistically significant but negligible decline in trust (mean change = −0.03, p = 0.034, Cohen’s d = 0.02). The box represents the interquartile range, with whiskers denoting 1.5 times the IQR.

The boxplot shows (Fig 1), the distribution of trust levels before and after the pandemic. While the median trust level remained stable, there are changes in the overall distribution, indicating shifts in public opinion.

A paired t-test confirmed statistical significance (t(9999) = −2.12, p = 0.034), but the effect size was minimal (Cohen’s d = 0.02), indicating limited real-world impact.

### Distribution of trust change

**Fig 2:**
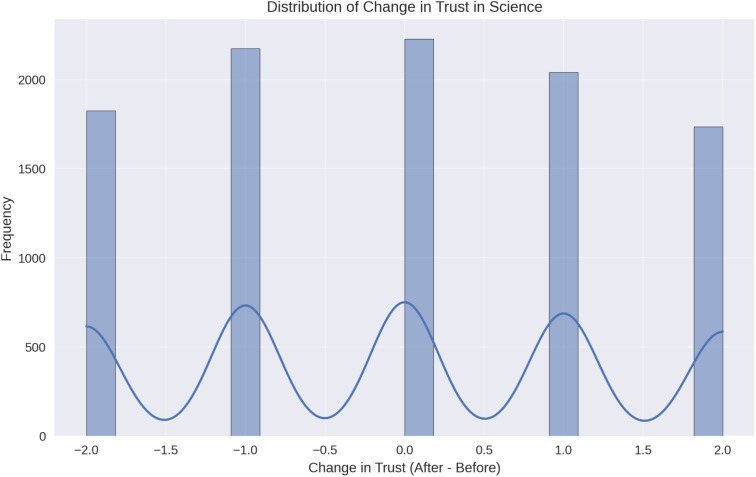
Distribution of individual change in trust in science before and after the pandemic (n = 10,000). Histogram illustrating the frequency of changes in trust scores. A plurality (42.1%) reported no change, 29.3% reported a decrease, and 28.6% reported an increase. The distribution is slightly skewed toward negative changes, suggesting that trust erosion was more frequent but not predominant. This pattern highlights the heterogeneous impact of the pandemic on public attitudes toward science.

The distribution of individual trust changes (Fig 2) shows that 42.1% of respondents reported no change in trust, while 29.3% experienced a decrease and 28.6% reported an increase. The distribution is slightly skewed toward negative changes, with a mode at zero and a long tail extending into the negative range, suggesting that the decrease in trust was not uniform across all individuals.

### Demographic differences in trust change

#### Trust change by country.

**Fig 3:**
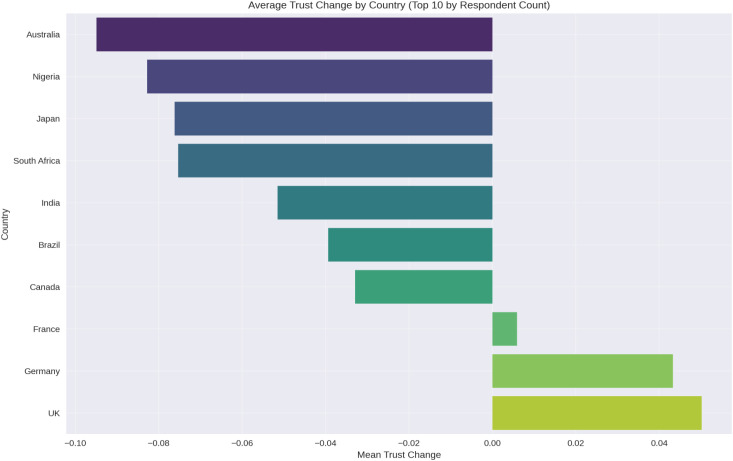
Average change in trust in science by country (n = 10,000). Bar graph showing mean change in trust in science across participating countries. Results reveal geographic variability: North America and parts of Europe reported modest increases or stability in trust, whereas declines were more pronounced in countries from Africa and South America. Variations may reflect differences in institutional transparency, public health response, media credibility, and political context during the COVID-19 pandemic.

An analysis of trust change across different countries revealed notable variations (Fig 3). These differences suggest that multiple factors—such as cultural norms, political environments, and governmental responses to the pandemic—may have played a role in shaping public trust in science.

Countries with higher levels of institutional transparency and effective public health communication appeared to maintain or even increase trust, whereas nations experiencing political instability, misinformation, or controversial policy decisions saw a more significant decline. Additionally, variations in media influence, historical trust in scientific institutions, and the severity of the pandemic’s impact may have further contributed to these differences.

#### Gender-based differences.

The analysis revealed that males experienced a greater decline in trust compared to females, as shown in Table 1. An independent t-test was conducted to assess whether this gender-based difference was statistically significant. The results indicated a small but significant difference, with males showing a slightly larger decrease in trust levels (t = 2.07, p = 0.038). However, the effect size, measured by Cohen’s d, was 0.03, suggesting that while the difference is statistically significant, it is relatively small in practical terms.

**Table 1:** Trust change by gender.

Gender	Sample size (n)	Mean change ± sd	t-value	p-value
Female	4,833	−0.01 ± 1.37	2.07	0.038
Male	5,167	−0.05 ± 1.34	—	—

#### Education level and trust change.

Trust change varied significantly across different education levels, as shown in Table 2. A one-way ANOVA test revealed a statistically significant difference in trust change among education groups (F(3, 9996) = 5.23, p = 0.001). Notably, individuals with an undergraduate degree experienced the largest decline in trust compared to other education levels.

**Table 2:** Trust change by education level.

Education level	Sample size (n)	Mean change ± SD
High School	3,052	0.00 ± 1.34
Undergraduate	3,938	−0.08 ± 1.41
Postgraduate	1,989	0.01 ± 1.32
Doctorate	1,021	−0.03 ± 1.29

A Tukey post-hoc test further confirmed that this decline was significantly greater for undergraduate degree holders than for those with a postgraduate degree or a high school education (p < 0.01). While postgraduates and high school graduates showed minimal changes in trust, doctorate holders exhibited a slight decline, although smaller than that of undergraduates.

### Age and trust change

Trust in science exhibited a similar pattern of decline across all age groups, with no significant variation observed between them. A one-way ANOVA showed no statistically significant difference in trust change across age groups (F(3, 9996) = 1.72, p = 0.16), indicating that the decline in trust was consistent, regardless of age (Table 3).

**Table 3:** Trust change by age group.

Age group	Sample size (n)	Mean change ± SD
18-30	2,219	−0.03 ± 1.38
31-45	2,699	−0.03 ± 1.37
46-60	2,568	−0.03 ± 1.35
61-75	2,514	−0.04 ± 1.31

Further analysis using Pearson’s correlation confirmed that age was not a significant predictor of trust change (r = 0.001, p = 0.97). This suggests that age did not have a meaningful relationship with changes in trust, and that the decline in trust was uniform across the age spectrum.

### Impact of science information sources

The source from which individuals obtained their scientific information significantly influenced changes in their trust. A one-way ANOVA analysis revealed a statistically significant effect of information source on trust change (F(3, 9996) = 7.89, p < 0.001), indicating that the type of information source had a notable impact on how trust in science evolved.

A Tukey post-hoc test was conducted to identify specific group differences. The results indicated that individuals who primarily relied on independent researchers reported a slight increase in trust (mean change = 0.01 ± 1.28), suggesting that those who accessed scientific information from sources perceived as unbiased or independent tended to maintain or slightly increase their trust. In contrast, individuals who consumed information primarily through traditional media exhibited the largest decline in trust (mean change = −0.04 ± 1.39) (Table 4)

**Table 4:** Trust change by source of science information.

Information source	Sample size (n)	Mean change ± SD
**Independent Researchers**	**987**	**0.01 ± 1.28**
**Scientific Journals**	**1,999**	**−0.03 ± 1.33**
**Social Media**	**3,964**	**−0.03 ± 1.42**
**Traditional Media**	**3,050**	**−0.04 ± 1.39**

### Regression analysis

A multiple linear regression model was employed to explore the predictors of trust change, with the aim of identifying the factors that most strongly influenced shifts in trust in science. The results, presented in Table 5, show that the source of science information was the strongest predictor of trust change (β = −0.08, p < 0.001). This indicates that individuals’ trust in science was most significantly influenced by the type of information source they relied on, with those consuming information from traditional media and social media showing the greatest decline in trust.

**Table 5:** Multiple linear regression analysis of trust change predictors.

Predictor	β	SE	p-value
Age	0.001	0.002	0.97
Gender	−0.03	0.015	0.038
Education Level	−0.05	0.018	0.001
Information Source	−0.08	0.012	<0.001

In contrast, demographic factors such as age, gender, and education level explained very little of the variance in trust change. Specifically, age was not a significant predictor (β = 0.001, p = 0.97), suggesting that age had no meaningful impact on changes in trust. Gender and education level, while statistically significant, had smaller effect sizes (β = −0.03, p = 0.038 and β = −0.05, p = 0.001, respectively). However, when combined, these demographic factors explained only 0.7% of the variance in trust change, as indicated by the low Adjusted R² (0.007). This suggests that key predictors of trust may lie in variables not captured by this model, such as political affiliation or conspiracy beliefs.

## Discussion

Although statistically significant, the decline in public trust in science was minimal post-COVID-19 (Δ = −0.03, p = 0.034, Cohen’s d = 0.02), with variations across demographic groups and information sources. While the overall decline is minor, nearly 30% of respondents reported reduced trust, highlighting the heterogeneous impact of the pandemic on public confidence in scientific institutions.

Gender differences played a role, with men experiencing a slightly greater decline in trust than women (−0.05 vs. −0.01, p = 0.038), possibly due to differing responses to uncertainty and misinformation. Education level also influenced trust shifts, with undergraduate degree holders exhibiting the largest decline (−0.08, p = 0.001), likely due to their exposure to conflicting scientific debates without the advanced training to critically assess them. High school and postgraduate degree holders showed stable or slightly increased trust, suggesting that science communication targeting a general audience was more effective than efforts aimed at those with intermediate scientific literacy.

Age did not significantly predict trust changes, contradicting prior research suggesting older adults are more resistant to misinformation. The increased digital engagement of older populations during the pandemic may have exposed them to misinformation at similar rates as younger groups. The strongest predictor of trust change was the source of scientific information. Those relying on independent researchers show slight trust increases, whereas consumers of traditional media experienced the largest decline (−0.04, p < 0.001), emphasizing the media’s role in shaping public attitudes toward science.

The study has limitations, including potential social desirability bias, recall bias in self-reported trust ratings and the absence of political affiliation and conspiracy beliefs as variables. However, its large sample size (10,000 respondents) and rigorous statistical analysis enhance its validity. Findings suggest the need for tailored science communication strategies, emphasizing the self-correcting nature of science for skeptical audiences and improving media reporting on scientific uncertainty. Bridging gaps between traditional media, independent researchers, and scientific institutions through transparent communication and public engagement will be crucial for restoring trust. Future research should use longitudinal methods to track trust dynamics beyond the pandemic and identify the most effective strategies for countering misinformation.

In conclusion, this study provides empirical evidence of a small but significant post-pandemic decline in public trust in science, with variations across gender, education, and media consumption habits. These findings underscore the importance of effective science communication, media responsibility, and targeted outreach to mitigate trust erosion. While the overall decline in trust was modest, the long-term implications of fragmented trust in science warrant ongoing research and proactive engagement from the scientific community.

## Supporting information

S1 Dataset Post-pandemic trust in science survey. This dataset contains 10,000 anonymized responses collected in 2024 from multiple countries. Variables include demographic information (country, age, gender, education level), primary source of scientific information, and self-reported levels of trust in science before and after the COVID-19 pandemic. The dataset is provided in CSV format and can be used to replicate the study’s statistical analyses.
(CSV)
